# Neutrophil Extracellular Traps in ANCA-Associated Vasculitis and Interstitial Lung Disease: A Scoping Review

**DOI:** 10.3390/life12020317

**Published:** 2022-02-20

**Authors:** Miriana d’Alessandro, Edoardo Conticini, Laura Bergantini, Paolo Cameli, Luca Cantarini, Bruno Frediani, Elena Bargagli

**Affiliations:** 1 Respiratory Diseases Unit, Department of Medical and Surgical Sciences & Neurosciences, University of Siena, 53100 Siena, Italy; dalessandro.miriana@gmail.com (M.d.); laurabergantini@gmail.com (L.B.); paolocameli88@gmail.com (P.C.); bargagli2@unisi.it (E.B.); 2 Rheumatology Unit, Department of Medicine, Surgery & Neurosciences, University of Siena, 53100 Siena, Italy; cantarini@unisi.it (L.C.); fredianibruno60@gmail.com (B.F.)

**Keywords:** NET, ANCA-associated vasculitis, interstitial lung disease, pulmonary fibrosis

## Abstract

Background: Deregulated neutrophil extracellular traps (NETs) formation is implicated in various diseases, including ANCA-associated vasculitis and pulmonary fibrosis (PF). Lung involvement is frequent in AAV, and interstitial lung diseases (ILDs) are strongly related to MPO-ANCA positivity and mainly reported in microscopic polyangiitis. The association between AAV and ILD is a strong indicator of poor prognosis and limited survival. Neutrophils, ANCA and NET interplay in PF development in AAV. This study aimed to review the literature concerning the implications of NET in lung fibrogenesis specifically focused on AAV associated with ILD, and the potential of NET as a theranostic marker. Methods: Through scoping review methodology, we used a descriptive thematic analysis to understand the pathogenic role of NETs in patients with AAV and pulmonary fibrosis and their further role as a theranostic marker of this disease. Results: The implications of NET in the pathogenesis of AAV and ILD, as well as an association between these two diseases, have been identified, but the underlying pathophysiological mechanisms are still unknown. The pharmacological or genetic inhibition of NET release reduces disease severity in multiple inflammatory disease models, indicating that NETs are potential therapeutic targets. In this regard, despite the lack of clinical data, we may hypothesise that an optimal management of AAV-ILD patients would require not only B-cells targeted therapy, but also NETs inhibition. Conclusion: Preliminary findings seem to display a lack of efficacy of traditional immunosuppressants, such as Rituximab, in this subset of patients, while to date no patients suffering from a definite ILD have been enrolled in clinical trials. Further insights would be provided by their employment, as a combination treatment, in common clinical practice. Although we can imagine that the inhibition of NETs in patients with AAV-ILD could reduce severity and mortality, we still lack the scientific basis that could improve our understanding of the disease from a molecular point of view.

## 1. Introduction

Innate immune cell responses have been linked to fibroblast and myofibroblast biology and fibrogenesis [1]. In particular, the alteration of immune cells (macrophages, neutrophils) in pulmonary fibrosis (PF) significantly impairs antimicrobial host defence functions, promoting susceptibility to infection [2,3].

Pulmonary fibrosis is a chronic progressive respiratory condition determined by an exaggerated dysregulated tissue repair response triggered by infections, cigarette smoke, environmental and occupational pollutants, obesity, diabetes mellitus, gastroesophageal reflux, pulmonary hypertension, chemotherapy and autoimmune disorders (including rheumatoid arthritis, scleroderma, Sjögren’s syndrome and vasculitis) [4,5,6,7,8,9,10,11,12,13,14]. However, it can also manifest without any known aetiology, as in the case of idiopathic pulmonary fibrosis (IPF), the most common and lethal fibrotic interstitial lung disease (ILD) [15,16].

Neutrophils are not only antibacterial effectors, but also influence their tissue environment by releasing proteases, oxidants, cytokines and chemokines [17]. They produce elastase which activates tumour growth factor-beta (TGF-β) and recruits inflammatory cells to the lung, thereby promoting PF [18,19,20].

Neutrophil-mediated injury plays a key role in interstitial fibrosis and abnormal lung repair in IPF [21,22,23]. Several studies have investigated the contribution of neutrophils to the development of fibrosis in terms of extracellular matrix (ECM) homeostasis, which regulates the balance between metal proteinases and metal proteinase inhibitors [24]. Neutrophils are reported to be increased in bronchoalveolar lavage (BAL) fluid from IPF patients and BAL neutrophil percentage has been linked to early mortality [20,25].

Neutrophils react against pathogens through three mechanisms including NETosis [26], which is a form of cell death induced by neutrophils through the release of neutrophil extracellular traps (NETs), which are fibrous DNA structures that protrude from the membranes of activated neutrophils [27,28]. Under physiological conditions, neutrophils release NETs as an antimicrobial defence. However, sterile stimuli, such as phorbol 12-myristate 13-acetate (PMA), monosodium urate and calcium pyrophosphate dehydrate crystals, may also induce NET formation [29]. It was recently demonstrated that neutrophils may remain viable and functional even after NET extrusion, principally when the scaffold is composed of mitochondrial DNA [30].

Two main processes trigger NET formation through chromatin decompensation: (i) the disintegration of nuclear and granular membranes and the release of enzymes, such as neutrophil elastase and myeloperoxidase (MPO) which alter the neutrophil cytoskeleton and decompensate chromatin [31]; and (ii) the enzyme peptidyl arginine deiminase IV (PAD4), which converts arginine residues into citrulline residues via nicotinamide adenine dinucleotide phosphate (NADPH)-oxidase (ΝOΧ), leading to histone deamination, loss of positive charge and chromatin decompensation [32].

In addition to the above, autophagy is also involved in NET formation through mTOR pathway inhibition, which increases autophagosome formation and accelerates NET formation [33,34].

Deregulated NET formation is implicated in various diseases, including autoimmune and autoinflammatory diseases, vein and arterial thrombosis, acute myocardial infarction, cancer and fibrosis [35,36]. Depending on the pathophysiological context of the disease, NETs are composed of different proteins that may account for their differential contribution to disease pathogenesis and phenotype [37,38]. In rheumatoid arthritis [39,40], anti-neutrophil cytoplasmic antibody (ANCA)-associated vasculitis (AAV) [41,42] and systemic lupus erythematosus [43,44], NETs are rich in immunogenic autoantigens and damage-associated molecular patterns, while NETs enriched in IL-17 promote fibrosis in ILD [45].

Here, we reviewed the literature concerning the implications of NETs in lung fibrogenesis. We also specifically focused on AAV associated with ILD and the potential of NET as a theranostic marker. There is growing interest in the involvement of NETs in inflammation and in the ensuing fibrosis.

## 2. Methods

A scoping review methodology was used according to the scoping review protocol [46]. We used descriptive thematic analysis to understand the pathogenic role of NETs in patients with AAV and pulmonary fibrosis and their further role as a theranostic marker of this disease. This article conforms with the Scale for Assessment of Narrative Review Articles (SANRA) guidelines [47].

### 2.1. Eligibility Criteria

The inclusion criteria were peer-reviewed, empirical or perspective papers with (a) relevance to the study topic: neutrophil extracellular traps, ANCA-associated vasculitis, pulmonary fibrosis and interstitial lung diseases; (b) language: English; (c) type of journal: preferences for journals related to pneumology and rheumatology with full text; (d) type of study: original article and multicentre study. Studies were excluded if (a) they were not relevant to the study topic; (b) they were written in languages other than English; (c) they did not adequately report objectives and conclusions; (d) they did not carry full text; (e) they were reviews, systematic reviews, meta-analyses, case reports, case series, correspondence or clinical trials.

### 2.2. Information Sources and Search

A systematic search of the literature was conducted in the PubMed online database. The Boolean search syntax was: neutrophil extracellular traps AND (“pulmonary fibrosis” OR “interstitial lung disease” OR “pulmonary fibrosis, vasculitis” OR “ANCA-associated vasculitis”). The search was limited to the English language and full texts. We did not include the grey literature (e.g., official reports from international organisations).

### 2.3. Selection Process

The abstract and title screenings and the full-text assessments were made against the eligibility criteria and were conducted by two independent reviewers (M.d. and E.C.) following pilot screenings with at least 80% agreement, and overseen by the leading review author (M.d.). Any discrepancies were resolved through consensus or the leading author’s input.

### 2.4. Data Charting and Items

**Figure 1:**
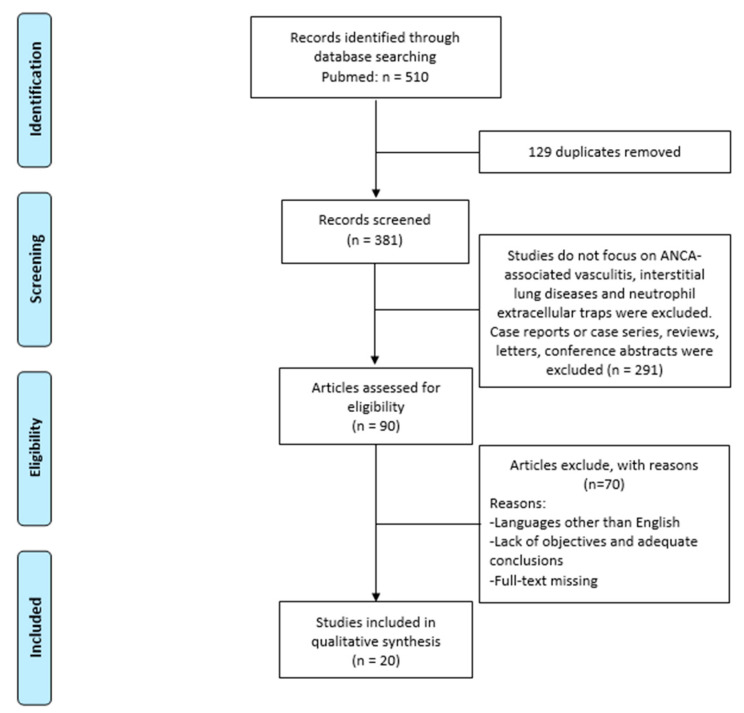
Flowchart of the selected articles used for the review.

Applying a coding structure designed by members of the research team, one author (M.d.) extracted the formal data elements (publication type, sources, geographies, objectives and main findings) with a random 5% verified by another author (L.B.). Regarding the included literature content, three independent reviewers (B.F., L.C. and E.B.) extracted the text quotations on (1) NETs in lung fibrosis or (2) NETs in vasculitis or (3) NETs in AAV lung fibrosis. These independent extractions were later paired for qualitative data synthesis, which was also independently informed by a brief synthesis of the papers by two reviewers. Then, the content of these extractions was merged (i.e., presented as the combined extractions of all reviewers) and the reviewers’ syntheses of the papers were combined. Figure 1 shows a flowchart of the selected articles.

## 3. Results

### 3.1. Synthesis of the Results: Descriptive Data

The search yielded entire texts. We selected 20 studies: 6 included analyses of NETs in pulmonary fibrosis (including ILD, COVID-19 and post-COVID-19 fibrosis); 1 of which proposed the involvement of NETs in the pathogenesis of IPF; 11 analysed the role of NETs in the pathogenesis of AAV (including GPA, MPA and EGPA); and 3 articles suggested the potential of NET inhibition as a therapeutic strategy for human fibrosis diseases. All these articles presented original data, albeit sometimes in experimental models.

### 3.2. Quality Assessment by SANRA Guidelines

The results of SANRA are reported in Table 1. All 60 ratings (3 raters × 20 manuscripts) were used for statistical analysis. The mean sum score across all 20 manuscripts was 8.0 out of 12 possible points (SD 0.0, range 7–8, median 8). The highest scores were rated for item 5 (scientific reasoning) (mean 1.8; SD 0.44); item 1 (justification of the article’s importance for the readership) (mean 1.7; SD 0.46); item 6 (appropriate presentation of data) (mean 1.62; SD 0.49); and item 2 (statement of concrete aims or formulation of questions) (mean 1.5; SD 0.51)—whereas item 4 had the lowest score (mean 1.3; SD 0.46). Item 3 had a score of 0 because one of the eligibility criteria excluded reviews of the literature.

### 3.3. NETs in Fibrotic Interstitial Lung Diseases

A pathogenic role of NETs has been described for many human diseases and the detrimental effects of excessive release of NETs are particularly significant for lung diseases, since this expands the alveoli, causing lung injury in asthma, chronic obstructive pulmonary disease, ILD and post-COVID-19-fibrosis, etc. [48,49,50,51,52,53]. Moreover, NETs and their associated molecules can directly induce epithelial and endothelial cell death [54]. NET formation must therefore be closely regulated to prevent NET-mediated tissue damage [31]. Recent therapies targeting NETs in lung diseases include the disintegration of DNA with human recombinant DNase, and the neutralisation of NET proteins with anti-histone antibodies and protease inhibitors [55]. ILD is often associated with specific environmental exposure or an underlying connective tissue disease [31]. An increase in circulating cell-free NETs and plasma LL-37 (a NET component) as well as lower DNase activity were reported in autoimmune rheumatic diseases associated with ILD, suggesting that prolonged exposure to NETs is involved in the development of ILD [56].

In vitro, NETs have been demonstrated to promote the activation of lung fibroblasts and their differentiation into a myofibroblast phenotype. Interestingly, these fibrotic effects were significantly less frequent after the degradation of NETs with DNase [45]. Confirming these findings, the detection of NETs close to alpha-smooth muscle actin-expressing fibroblasts was demonstrated in biopsies from patients with fibrotic ILD [31], probably mediated by the directed effect of neutrophil elastase [57]. Accordingly, a neutrophil elastase inhibitor attenuated pulmonary fibrosis in a murine model through the inhibition of TGF-β1 and inflammatory cell recruitment to the lungs [58]. Some authors demonstrated that the release of NETs by neutrophils treated with fibrosis-related agents (i.e., bleomycin) or with generic NET inducers (i.e., PMA) induced the activation of fibroblasts and their differentiation into the myofibroblast phenotype [59]. Altogether, these findings point to a key role of NETs in the development and progression of PF. Recently, Suzuki and colleagues identified NETs in the alveolar and interstitial lung space of mice undergoing bleomycin-induced lung fibrosis, which was suppressed by a pan-PAD inhibitor, demonstrating that Padi4 gene knockout in mice led to the alleviation of bleomycin-induced NETs and pulmonary fibrosis; this finding suggests that this pathway could be useful as a therapeutic target in the treatment of pulmonary fibrosis [60].

### 3.4. NETs in ANCA-Associated Vasculitis

ANCA-associated vasculitis (AAV) is life-threatening small vessel inflammation, affecting the kidneys, upper and lower airways, skin and central and peripheral nervous systems [61,62]. Three different clinical types of AAV can be distinguished: granulomatosis with polyangiitis (GPA); microscopic polyangiitis (MPA); and eosinophilic granulomatosis with polyangiitis (EGPA) [63]. Most evidence on the role of ANCA in the pathophysiology of these diseases comes from animal models [64]. ANCA participates in the migration and activation of neutrophils causing ROS and protease release through cell degranulation [65]. Various stimuli activate neutrophils, causing damage to the endothelium with positive feedback from activated endothelial cells that recruit monocytes and T cells, releasing pro-inflammatory mediators [19,21,24]. Monocytes express myeloperoxidase (MPO) and proteinase 3 (PR-3) on their surfaces, and ANCA binds to these molecules, activating monocytes and secreting cytokines (such as IL-8 and monocyte chemoattractant protein-1). In the case of MPO-AAV and PR3-AAV, there is a loss of immunological tolerance to neutrophil enzymes MPO and PR3, which in turn generates ANCA- and MPO-specific T cells. Neutrophils activated by ANCA induce cell cytotoxicity and cause direct tissue injury, thus inducing systemic vasculitis. In up to 90% of patients, ANCA is directed against MPO and PR-3 in neutrophil granules and macrophage lysosomes [66].

It has been reported that neutrophil priming and activation via a complement system (C5a and C5aR interactions) exteriorise MPO (or PR3) on their cell surfaces which MPO-ANCA (or PR3 ANCA) binds to. These neutrophils are home to the microvasculature of the glomeruli involving selectins which play a role in reducing the speed of the neutrophils in the circulation which allows it to roll. The second signal comes from the neutrophil surface integrins β1 and β2 which interact with the ICAM-1 and ICAM-2 ligands on the inflamed endothelium; then, the rolling neutrophils slow down and crawl along the endothelium mediated by integrins to the site of transmigration from the capillaries into the interstitium. Neutrophils either degranulate or release NETs, causing direct injury through the release of enzymes, ROS and proteases [67]. Excess neutrophils, MPO-specific T cells, and macrophages (via a delayed type of hypersensitivity mechanism) generate a vicious cycle of injury which causes glomerular injury.

NETs contain proinflammatory proteins and are thought to directly contribute to vessel inflammation by damaging endothelial cells and by activating the complement system and indirectly contribute to vessel inflammation by acting as a link between the innate and adaptive immune system through the generation of PR3- and MPO-ANCA.

Interestingly, increased levels of NETosis-derived products (or NET remnants) in circulation have been reported in a small number of patients with active vasculitis [68]. Soderberg and colleagues analysed for the first time increased levels of NET remnants in a larger cohort of AAV patients with active disease and an inverse correlation between such levels and ANCA at least during remission [69].

The production and release of NETs by activated neutrophils have also recently been implicated in the pathophysiology of AAV [70,71,72,73,74,75], although the precise mechanisms are not yet known. It is speculated that aberrant NETs could be autoantigens for ANCA underlying the development of MPA, and that the in vivo inhibition of NETs could be a therapeutic strategy for the disease [76]. Kimura and colleagues devised a mouse model of MPA, induced by the administration of a novel extract from Candida albicans and lacking phosphoinositide 3-kinase gamma (PI3K-gamma), and demonstrated the accumulation of NETs in vivo, the elevation of ANCA titres, small-vessel vasculitis and crescentic glomerulonephdritis [76]. The blockade of PI3K-gamma reduced these abnormalities, indicating its potential as a therapeutic molecule [76]. A group of Japanese researchers demonstrated the efficacy of intravenous immunoglobulin therapy in the inhibition of NET formation induced by PMA in vitro, improving the development of MPO-AAV [77]. A role of platelets stimulated via Toll-like receptor (TLR) pathways in NET formation was recently reported, although the underlying mechanism is unclear [78]. Matsumoto et al. cultured platelets from AAV patients, treating them with a TLR-agonist CXCL4 and/or with anti-CXCL4 antibody. They showed that neutralising the anti-CXCL4 antibody significantly inhibited NET formation [79].

Interestingly, some authors showed that neutrophils from AAV patients are less susceptible to apoptosis [80], suggesting that these neutrophils are more prone to other forms of cell death. Indeed, in vitro studies have shown that neutrophils from AAV patients spontaneously release more NETs than neutrophils from healthy blood donors [69,81,82]. These populations of neutrophils were reported by Greyson et al. as low-density granulocytes (LDGs) and have been proposed to be the major source of NETs in AAV [81]. However, the same authors showed that normal-density neutrophils from AAV patients spontaneously released more NETs than normal-density neutrophils from healthy blood donors.

Thus, future in vitro studies of NETs need to take account that during the neutrophil’s isolation procedures from peripheral whole blood, LDGs will not be included because they will be found in the fraction of peripheral blood mononuclear cells.

Few papers reported the implication of NETs in two clinically and pathologically different diseases, AAV and SLE. Van Dam and colleagues compared the mechanisms of NET formation and the composition of NETs in AAV and SLE. They revealed the differences between AAV and SLE in terms of NET formation and provided a better understanding of the pathophysiologic role of NETs in these different autoimmune diseases [83].

Alternative complement pathway activation plays a role in the pathogenesis of AAV [84]; its inhibition, mainly targeting C5a component, is of interest due to its role in neutrophil activation and migration [85]. This provides a rationale for using avacopan (CCX168), an oral C5a inhibitor recently approved by the FDA for the treatment of GPA and MPA, on the basis of an ADVOCATE trial which displayed a non-inferiority to prednisone with respect to remission at week 26 and superiority with respect to sustained remission at week 52 [86].

### 3.5. NETs in ANCA-Associated Vasculitis with Pulmonary Fibrosis

Pulmonary manifestations are frequent in AAV [87]. They range from nodules and transient infiltrates, which are more frequent in GPA and EGPA [88], respectively, alveolar haemorrhage, which can be the presenting sign of MPA, to ILD, which is mainly reported in MPA and strongly related to MPO-ANCA positivity [89]. The association between AAV and ILD is a strong indicator of poor prognosis and limited survival. Early detection of this association is crucial to the management of these patients, although no specific treatments have been proposed to date.

Pulmonary fibrosis in patients with MPA can lead to chronic respiratory failure and increased risk of potentially fatal acute exacerbations. The exact mechanisms involved in fibrosis development in AAV patients have been suggested to include the relation between the release of NETs [90] and the development of PF, although the pathobiological pathways are far from clear. It is speculated that neutrophils, ANCA and NETs interact to cause PF in AAV [91]: activated neutrophils release NETs that reach the endothelium or interstitium, participating in all stages of the development of PF. This assumption is in line with the general pathogenetic model of CTD-ILD proposed by Wells and Denton [92]: in CTD-ILD patients, the lung disorder develops in three stages: initiation, progression and failed resolution. After inflammation and endothelial activation, which result in epithelial damage, fibroblasts are recruited, and their proliferation and activation mark the transition to the progression phase. The specific molecular pathways of ILD development in AAV are still largely unknown, however, Negreros and Flores-Suárez postulated that NET-associated proteins (NAPs) may act as mediators in the development of fibrosis through the modification of ECM composition, increasing levels of collagen, osteopontin and fibronectin [91]. NET-associated proteins increase the number and activity of fibroblasts and profibrotic cytokines and inhibited the fibroblast apoptosis. The more NETs persist in the interstitium, the more NAPs affect fibroblasts. In the event of failure to remove NETs due to their resistance to degradation and low DNAse I activity in serum, AAV develops, and the effects of NAPs are maintained [91].

A few months ago, Chirivi and colleagues described the inhibition of NETs with therapeutic anti-citrullinated protein antibody (tACPA) for the first time in mice models. NET-mediated inflammatory diseases (including inflammatory arthritis and pulmonary fibrosis) enable eliminating the noxious triggers that lead to persistent inflammation and tissue damage [93].

In the initial stage of lung fibrosis in patients with AAV, any modifications of the interstitial microenvironment and resident cell behaviour exerted by neutrophils and NETs in the interstitial lung space have often been overlooked. Although some authors have demonstrated the effects of NETs on lung fibroblast proliferation and differentiation [91], the mechanisms by which NETs exert these effects remain unclear.

## 4. Conclusions

The implications of NETs in the pathogenesis of AAV and ILD, as well as an association between these two diseases, have been identified, however, the underlying pathophysiological mechanisms are still unknown. NETs contain proinflammatory proteins and are thought to directly contribute to vessel inflammation by damaging endothelial cells and by activating the complement system and indirectly contribute to vessel inflammation by acting as a link between the innate and adaptive immune system through the generation of PR3- and MPO-ANCA.

Although not strictly related to a complement pathway nor to the direct neutrophils’ activation, it is worth mentioning that several other cytokines seem to play a crucial role in the pathogenesis of AAV. In this regard, serum IL-1, IL-32 and IL-6 [94,95,96] seem to be elevated in patients suffering from AAV and directly correlate with disease activity [97], namely skin vasculitis in patients with GPA and high levels of ANCA-PR3 [95], also displaying a direct correlation with the risk of relapse [98] among the patients treated with anti-CD20 agents.

On the other hand, despite such interesting insights which may have paved the way for the use of Tocilizumab in AAV [96,97], anti-IL-6 agents have provided scanty and disappointing data [99] and their real-life use is restricted to case reports with doubtful diagnosis [100] or small case series.

The pharmacological or genetic inhibition of NET release reduces disease severity in multiple inflammatory disease models, indicating that NETs are potential therapeutic targets.

Despite the lack of clinical data, we may hypothesise that an optimal management of AAV-ILD patients would require not only B-cells’ targeted therapy but also NET inhibition.

Preliminary insights from avacopan registrative studies [101,102,103] suggest that the inhibition of a complement pathway, which is in turn related to NET activation, may be a promising option in the treatment of AAV. Nevertheless, the exact role of a complement pathway in the pathogenesis and disease activity of AAV is still matter of debate: while some papers have suggested that hypocomplementemia may be associated with a poorer renal outcome [104,105], other have not evidenced any difference in terms of C3 and C4 serum levels in AAV patients when compared with subjects affected by other kidney diseases [106].

Preliminary findings seem to display a lack of efficacy of traditional immunosuppressants, such as Rituximab, in this subset of patients, while to date, no patients suffering from a definite ILD has been enrolled in clinical trials. Further insights would be provided by their employment, as a combination treatment, in common clinical practice.

Although it can be speculated that NETs in patients with AAV-ILD could reduce severity and mortality, a scientific basis that could improve our understanding of the disease from a molecular point of view is lacking.

## Figures and Tables

**Table 1:** SANRA score for quality assessment.

N°.	Title and Authors	Justification of the Article’s Importance for the Readership	Statement of Concrete Aims or Formulation of Questions	Description of the Literature Search	Referencing	Scientific Reasoning	Appropriate Presentation of Data	Total Score
1	Neutrophil Extracellular Traps Induce the Epithelial—n Mesenchymal Transition: Implications in Post-COVID-19 Fibrosis. Pandolfi L., Bozzini S., Frangipane V. et al., 2021	2	2	0	1	2	2	9
2	Therapeutic ACPA inhibits NET formation: a potential therapy for neutrophil-mediated inflammatory diseases. Chirivi R.G.S., van Rosmalen J.W.G., van der Linden M. et al., 2021	2	2	0	2	2	2	10
3	PAD4 Deficiency Improves Bleomycin-induced Neutrophil Extracellular Traps and Fibrosis in Mouse Lung. Suzuki M., Ikari J., Anazawa R. et al., 2020	2	1	0	1	1	2	7
4	Neutrophil extracellular traps infiltrate the lung airway, interstitial, and vascular compartments in severe COVID-19. Radermecker C., Detrembleur N., Guiot J. et al., 2020	1	2	0	2	2	1	8
5	Neutrophil extracellular traps activate lung fibroblast to induce polymyositis-related interstitial lung diseases via TLR9-miR-7-Smad2 pathway. Zhang S., Jia X., Zhang Q. et al., 2019	2	1	0	2	2	1	8
6	Regulation of *Pseudomonas aeruginosa*-Mediated Neutrophil Extracellular Traps. Skopelja-Gardner S., Theprungsirikul J., Lewis K.A. et al., 2019	1	1	0	1	2	2	7
7	Identification of a Novel HIF-1α-α_M_β_2_ Integrin-NET Axis in Fibrotic Interstitial Lung Disease. Khawaja A.A., Chong D.L.W., Sahota J. et al., 2020	2	2	0	1	2	1	8
8	Neutrophil extracellular traps promote differentiation and function of fibroblasts. Chrysanthopoulou A., Mitroulis I., Apostolidou E. et al., 2014	2	1	0	2	2	1	8
9	Excessive neutrophil extracellular trap formation in ANCA-associated vasculitis is independent of ANCA. Kraaij T., Kamerling S.W.A., van Dam L.S. et al., 2018	2	1	0	2	1	2	8
10	Neutrophil extracellular trap formation is associated with autophagy-related signalling in ANCA-associated vasculitis. Tang S., Zhang Y., Yin S.W. et al., 2015	2	1	0	1	2	1	7
11	Myeloperoxidase anti-neutrophil cytoplasmic antibody affinity is associated with the formation of neutrophil extracellular traps in the kidney and vasculitis activity in myeloperoxidase anti-neutrophil cytoplasmic antibody-associated microscopic polyangiitis. Yoshida M., Yamada M., Sudo Y. et al., 2016	1	2	0	1	2	1	7
12	Enhanced formation and disordered regulation of NETs in myeloperoxidase-ANCA-associated microscopic polyangiitis. Nakazawa D., Shida H., Tomaru U. et al., 2014	2	1	0	2	2	2	9
13	The presence of anti-neutrophil extracellular trap antibody in patients with microscopic polyangiitis. Hattanda F., Nakazawa D., Watanabe-Kusunoki K. et al., 2019	2	2	0	1	2	1	8
14	Necroptosis controls NET generation and mediates complement activation, endothelial damage, and autoimmune vasculitis. Schreiber A., Rousselle A., Becker J.U. et al., 2017	2	2	0	1	1	2	8
15	Intrinsically Distinct Role of Neutrophil Extracellular Trap Formation in Antineutrophil Cytoplasmic Antibody-Associated Vasculitis Compared to Systemic Lupus Erythematosus. Van Dam L.S., Kraaij T., Kamerling S.W.A. et al., 2019	2	2	0	1	1	1	7
16	Detection by flow cytometry of anti-neutrophil cytoplasmic antibodies in a novel approach based on neutrophil extracellular traps. Roitsch S., Gößwein S., Neurath M.F. et al., 2018	1	2	0	1	2	2	8
17	Neutrophil extracellular traps in neuropathy with anti-neutrophil cytoplasmic autoantibody-associated microscopic polyangiitis. Takeuchi H., Kawasaki T., Shigematsu K. et al., 2017	2	2	0	2	2	2	10
18	TIM-3 regulates the NETs-mediated dendritic cell activation in myeloperoxidase-ANCA-associated vasculitis. Su B., Mao X., Yin B. et al., 2021	2	1	0	1	2	2	8
19	Increased levels of neutrophil extracellular trap remnants in the circulation of patients with small vessel vasculitis, but an inverse correlation to anti-neutrophil cytoplasmic antibodies during remission. Söderberg D., Kurz T., Motamedi A. et al., 2015	2	1	0	1	2	1	7
20	Neutrophil-related and serum biomarkers in granulomatosis with polyangiitis support extracellular traps mechanism of the disease. Surmiak M., Hubalewska-Mazgaj M., Wawrzycka-Adamczyk K. et al., 2016	1	2	0	1	2	2	8

## Data Availability

The data presented in this study are available upon request from the corresponding author.
